# Saireito (114) Increases IC50 and Changes T-Cell Phenotype When Used in Combination with Prednisolone Therapy in Human Peripheral Blood Mononuclear Cells

**DOI:** 10.1155/2022/9738989

**Published:** 2022-02-28

**Authors:** Kyosuke Yamazaki, Anna Kiyomi, Shinobu Imai, Munetoshi Sugiura

**Affiliations:** Tokyo University of Pharmacy and Life Sciences, Tokyo 192-0392, Japan

## Abstract

Prednisolone (PSL), a type of corticosteroid used to treat autoimmune diseases, can increase the risk of infection and osteoporosis. Saireito (114), a Kampo medicine, has an immunosuppressive effect; with its use, the dose of steroids can be reduced. However, its mechanism when used with PSL is still unclear. We used peripheral blood mononuclear cells (PBMCs) from healthy adults to examine the effect of 114 and PSL treatment on PBMC proliferation, T-cell subsets, and cytokine production. PBMCs were cotreated with concanavalin A and 300 *μ*M 114 (either Tsumura & Co. (TJ) or Kracie Holdings (KR)) and 0.0001–1.0 *μ*M PSL for 96 h to create the T-cell mitogen. We then measured the PBMC proliferation; ratio of CD4^+^ T cells, CD8^+^ T cells, and T-follicular helper (Tfh) cells; and concentration of cytokines (TNF, IFN-*γ*, IL-6, IL-10, IL-17A, and IL-21). The proliferation of PBMCs was dose dependently suppressed in both the PSL and PSL + 114 groups (p  <  0.05). Combination therapy increased the IC_50_ in the PSL group (0.0947 *μ*M) by 2.02 and 1.64-fold in the PSL + TJ114 and PSL + KR114 groups, respectively. Both the PSL + 114 groups had an increased ratio of CD4^+^ T cells compared to the PSL group, with no effect on the ratio of CD8^+^ T and Tfh cells. Furthermore, the PSL + 114 groups showed increased IL-6 and IL-10 compared to the PSL monotherapy group, although the difference was not significant. There was no significant difference in the TNF, IFN-*γ*, IL-17A, and IL-21 concentrations between the PSL and PSL + 114 groups. The elevated IC_50_ with 114 cotreatment suggests diminished immunosuppressive action. Moreover, increased cytokine production by Th2 with 114 cotreatment suggests a restoration of T-cell balance in Th1-mediated autoimmune diseases. However, increased IL-6 suggests potential exacerbation of IL-6-mediated diseases, such as rheumatoid arthritis. Therefore, it is necessary to monitor these clinical parameters when using 114 in combination with PSL.

## 1. Introduction

Autoimmune diseases are characterized by abnormal immune function (overproduction of autoantibodies and immune cells) that attack a person's own healthy cells and tissue [1]. Various drugs are currently used to treat autoimmune diseases, including disease-modifying antirheumatic drugs (DMARDs), corticosteroids, nonsteroidal anti-inflammatory drugs (NSAIDs), anticytokines, and other similar medications [2]. Although prednisolone (PSL), a type of corticosteroid, is widely used in Japan, patients experience clinically significant adverse reactions, such as osteoporosis, immune deficiency, psychiatric disorders, adrenal gland dysfunction, increased susceptibility to infection, and hyperglycemia [3]. In addition, since the risk of adverse reactions correlates with increased corticosteroid dosage and duration of administration, it is important to minimize the dose required to regulate disease activity [4].

Over centuries, Kampo medicine has become well established as a result of the types, quantities, and combinations of natural medicines. In contrast to corticosteroids, Kampo medicine generally has few adverse effects. The increased safety is a major reason over 80% of doctors in Japan use it for routine care in combination with Western medicine [5]. This includes treatment for autoimmune diseases, with reports showing that treatment for rheumatoid arthritis (RA) is effective when using immunosuppressants (such as corticosteroids) in combination with Kampo medicine [6]. However, the mechanisms and effect of the combined use of Kampo medicine and immunosuppressants on the immune system are still unclear.

Saireito (denoted 114) is a Kampo medicine that is used in combination with corticosteroids (especially PSL) in the treatment of autoimmune diseases, such as RA, and is reported to have steroid-like immunosuppressive action [7]. In addition, due to its antigranulation effect, cotreatment with dexamethasone reportedly strengthens its anti-inflammatory action and reduces the dosage requirements of dexamethasone [8]. Additionally, the use of 114 in combination with PSL is expected to strengthen its immunosuppressive actions; however, this is yet to be confirmed.

T cells are a major contributor to autoimmune diseases. The T cells of the immune system are classified as cells that have a regulatory action, such as helper T cells (CD4^+^ T cells) and regulatory T cells (Treg), and cells that have effector functions, such as cytolytic T cells (CD8^+^ T cells). CD4^+^ T cells are classified as either Th1 or Th2, based on the pattern of cytokines they produce. For instance, Th1 cells mainly produce interleukin-2 (IL-2) and interferon-gamma (IFN-*γ*) and contribute to delayed hypersensitivity reactions, whereas Th2 cells produce IL-4, IL-5, IL-6, and IL-10 and contribute to the proliferation of B cells and production of antibodies [9]. Autoimmune diseases are thought to be the result of an imbalance between Th1 and Th2 [10] and may be classified as either Th1-or Th2 diseases. Th1-mediated autoimmune diseases are organ-specific diseases, such as multiple sclerosis and type-1 diabetes, while Th2-mediated autoimmune diseases are antibody-mediated diseases, such as systemic lupus erythematosus (SLE) and RA [11]. The primary treatment for autoimmune diseases includes a treatment strategy to correct the imbalance between Th1 and Th2 cells. In addition, recently discovered CD4^+^ T-cell subsets include Th17 cells, which produce IL-17. Th17 and IL-17 contribute to the etiology of multiple autoimmune-mediated inflammatory diseases [12]. T-follicular helper (Tfh) cells express the chemokine receptors CXCR5 and PD-1 while further inducing immunoglobulin production in B cells by producing IL-21 [13]. IL-21 can also be produced by Th17 and has various effects, ranging from Tfh and Th17 cell development, helper T-cell subset balance, production of B cells and immunoglobulins, and plasma cell differentiation, and is also associated with autoimmune diseases [14]. Studies have shown that excessive activation of Tfh cells induces SLE-like autoimmune diseases in mice [15] and humans [16]. For instance, Tfh cells are increased in the peripheral blood of patients with autoimmune diseases, such as RA and SLE, and the ratio of Tfh cells correlates with disease activity, organ damage, and autoantibody valence [17], such as anti-double-stranded DNA (dsDNA) and anti-cyclic citrullinated peptide antibodies (ACPA).

The same Kampo medicines are manufactured and sold by multiple pharmaceutical manufacturers, but the types and amounts of constituent crude drugs differ among those medicines depending on the pharmaceutical manufacturer. However, there are currently no clear standardized Kampo medicine guidelines. Therefore, it is equally important to examine whether there is a difference in efficacy between medicines from different manufacturers. This study compared the immunosuppressive efficacy of PSL monotherapy and PSL with 114 combination therapies (from two separate suppliers) in peripheral blood mononuclear cells (PBMCs) obtained from healthy donors. Altogether, we measured the ratio of CD4^+^ T cells, CD8^+^ T cells, and Tfh cells in PBMCs and the concentration of cytokines produced by Th1, Th2, Th17, and Tfh subsets.

## 2. Materials and Methods

### 2.1. Preparation of Medium and Reagent

We prepared the RPMI 1640 medium (Gibco, Waltham, MA, USA) by adding fetal bovine serum (FBS; Gibco), which was inactive for 30 min at 56°C, until it reached 10% (v/v), and then added penicillin-streptomycin (10,000 U/mL; Gibco) by adding 100 U/mL penicillin and 100 mg/mL streptomycin.

Concanavalin A (ConA; Seikagaku, Tokyo, Japan) was dissolved in RPMI 1640 medium at a concentration of 1 mg/mL.

PSL powder (molecular weight: 365.85; FUJIFILM Wako Pure Chemical, Osaka, Japan) was dissolved using dimethyl sulfoxide (FUJIFILM Wako Pure Chemical) to a final concentration of 10 *μ*M. We then performed serial dilutions using RPMI 1640 medium to produce 0.0001, 0.001, 0.01, 0.1, 0.25, 0.5, and 1 *μ*M (final concentration used in the cell culture system).

Tsumura (TJ114; Tsumura & Co, Tokyo, Japan) and Kracie Saireito (KR114; Kracie Pharmaceutical, Tokyo, Japan) were dissolved in RPMI 1640 medium to obtain a weighted granulated powder concentration of 10 mg/mL. We then centrifugated the mixture at 3,500 rpm for 10 min at 23°C. After centrifugation, the supernatant was filtered using a syringe with a 0.22 *μ*M membrane filter (AS ONE, Osaka, Japan) and used as the Kampo medicine reagent.

### 2.2. Subjects

Peripheral blood was obtained from healthy adults (two men and four women: average age, 23.7 ± 0.98) after receiving written informed consent. Furthermore, none of the subjects took medication from one week prior to the end of the study period. This study was conducted in accordance with the ethical standards of the World Medical Association (Declaration of Helsinki) and was approved by the Tokyo University of Pharmacy and Life Sciences Ethical Committee for Studies on Human Tissue (approval number: 20-5).

### 2.3. Isolation of PBMCs

We gently aliquoted 20 mL peripheral venous blood from the subjects (*n* = 6) (collected in heparin tubes) into lymphocyte separator liquid (Nacalai Tesque, Kyoto, Japan) and centrifuged it at 2,300 rpm for 20 min at 23°C. The PBMC layer was separated into a centrifuge tube using a Pasteur pipette and added 10 mL RPMI 1640 medium to cleanse the PBMCs. Thereafter, the cells were centrifuged at 1,500 rpm for 10 min at 23°C. The supernatant was removed, and the process was repeated once more. Finally, the PBMCs were suspended in 5 mL RPMI 1640 medium. Cell number was determined using a hemocytometer and a microscope. The average number of PBMCs from the six subjects was 2.396 × 10^7^ cells. The cell suspension was diluted with RPMI 1640 medium to reach a concentration of 1 × 10^6^ cells/mL. Cells were plated onto a 96-well plate, followed by addition of 10 *μ*L ConA as the T-cell mitogen (final concentration: 5 *μ*g/mL) and finally either 20 *μ*L PSL (final concentrations: 0.0001, 0.001, 0.01, 0.1, 0.5, and 1 *μ*M) or 20 *μ*L 114 (final concentration: 300 *μ*g/mL). The concentration of 114 was set according to the clinical concentration in human blood at steady-state (300 *μ*g/mL) [18].

As a blank for the 114-monotherapy group, we added 170 *μ*L PBMC suspension, 10 *μ*L ConA solution, and 20 *μ*L culture medium. As a blank for the PSL monotherapy and PSL + 114 groups, we added 170 *μ*L PBMC suspended solution, 10 *μ*L ConA solution, and 20 *μ*L dimethyl sulfoxide (final concentration: 0.4%). We then cultured the solutions at 37°C for 96 h in the presence of 5% CO_2_. Kampo medicine is thought to have weaker actions than Western medicine; therefore, we determined the incubation period of the solution to determine the culture duration without affecting the survival of the PBMCs.

### 2.4. Assessing the Inhibition of PBMC Proliferation

After cells reached confluence, we added 10 *μ*L cell counting kit-8 solution (Dojindo Laboratories, Tokyo, Japan) to each well and then further incubated the cells at 37°C for 3 h in the presence of 5% CO_2_. Afterwards, we used a multispectral scanning reader to measure the absorbance at a wavelength of 450 nm. The proliferation rate (%) of PBMCs in the presence of the drug at each PSL concentration was calculated by subtracting the absorbance (nm) of the respective blanks from that of PBMCs with treatments.

2.4.1. Flow Cytometry Analysis of CD4^+^ T Cells, CD8^+^ T Cells, and CD4^+^ CXCR5^+^PD-1^+^ T Cells (Tfh Cells)

For flow cytometry, we added 10 *μ*L PERCP-Cy5.5-labeled anti-human CD4, 5 *μ*L APC-Cy7-labeled anti-human CD8, 10 *μ*L Alexa Fluor 647-labeled anti-human CXCR5, and 5 *μ*L FITC-labeled anti-human CD279 (PD-1) monoclonal antibody solutions (all obtained from BD Pharmingen, San Jose, CA, USA), respectively, to the cultured cells from the previous section. We then incubated the solution at 37°C for 20 min in the presence of 5% CO_2_. After the incubation, we added 1 mL wash buffer (1% FBS in phosphate-buffered saline (PBS) (FUJIFILM Wako Pure Chemical)) and resuspended the solution. We then performed centrifugation at 1,300 rpm for 5 min at 23°C before washing the cells again. We resuspended the sediments obtained using a 400 *μ*L staining buffer and immobilized the cells. The cellular suspension was filtered through a nylon mesh (37 *μ*m holes; AS ONE, Osaka, Japan), transferred to a polystyrene tube for flow cytometry (FALCON, Aichi, Japan), and then dyed using a FACS Canto™ II (BD Pharmingen). The data analysis of 30,000 cells was performed using the software CELLQuest v3.1 (BD Pharmingen).

We gated the lymphocyte cell population using an FCS/SSC dot plot and collected data on lymphocytes. We then gated the CD4^+^ cells using PerCP-Cy5-5A as the CD4 detection wavelength to determine CD4^+^ T cells. We then gated CD8^+^ cells using APC-Cy7 as the CD8 detection wavelength to determine CD8^+^ T cells. After gating, we quartered the CD4^+^ T cells shown in the dot plot using Alexa Fluor 647 and FITC as fluorescence wavelengths for CXCR5 and PD-1. We then determined the positive cells of both CXCR5 and PD-1 (CD4^+^ CXCR5^+^ PD-1^+^ cells) to be Tfh cells.

Each T-cell subset was calculated using the following formulae:

CD4^+^ T cells were calculated by the number of CD4-stained cells / the number of lymphocytes *∗* 100; CD8^+^ T cells were calculated by the number of CD8-stained cells / the number of lymphocytes *∗* 100; Tfh cells were calculated by the number of CXCR5- and PD-1-stained cells / the number of CD4-stained cells *∗* 100.

### 2.5. Cytokine Measurements

We measured the concentrations of tumor necrosis factor (TNF), IFN-*γ*, IL-6, IL-10, IL-17A, and IL-21 in the supernatant collected from the cultured cells in previous sections using a Human Flex Set (BD Pharmingen, San Jose, CA, USA). We continuously added 50 *μ*L of each type of cytokine capture bead, 50 *μ*L detection reagent, and 50 *μ*L culture supernatant or the standard solution to each sample and then incubated the solution in the dark at 23°C for 3 h. The samples were then rinsed with wash buffer and centrifuged at 1300 rpm for 5 min at 23°C. After centrifugation, the supernatant was discarded, and 300 *μ*L wash buffer (in the Human Flex Set) was added to each sample before being resuspended. We then measured each sample using FACS Canto™ II. The cytokine concentration in each sample was determined by fluorescence intensity. We serially diluted the standard solution to create a standard curve for determining the concentration of each type of cytokine in the culture supernatant. We used the data analysis software FACP Array™ (BD Pharmingen, San Jose, CA, USA). Taking advantage of the fact that the absorbance value of WST is proportional to the number of living cells, the cytokine concentration corresponding to the number of cells at each concentration was corrected by dividing with the WST absorbance value.

### 2.6. Statistical Evaluation

We analyzed the significant difference between the blanks and the PSL monotherapy, 114-monotherapy, and PSL + 114 combination therapy groups using Dunnett's multiple comparisons test. In addition, we used Tukey's multiple comparison test to compare the PSL monotherapy and PSL + 114 combination therapy groups. For both tests, p  <  0.05 was taken as significantly different. We used GraphPad PRISM (v8.0; GraphPad Software, San Diego, CA, USA) for data analyses.

## 3. Results

### 3.1. Inhibition of PBMC Proliferation by Adding PSL and 114

**Figure 1:**
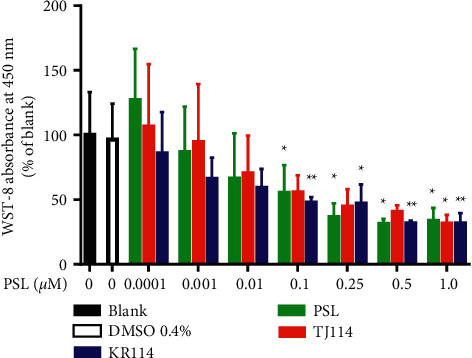
Inhibition of PBMC proliferation with PSL and 114 treatments. Proliferation of PBMCs stimulated by ConA after addition of PSL and 114 cultures. Each treatment was compared to its respective blank using Dunnett's multiple comparison test (^*∗*^*p* < 0.05; ^*∗∗*^*p* < 0.01). The PSL monotherapy group and the PSL + 114 combination therapy groups were compared using the Tukey-Kramer multiple comparison test (mean ± SD, n = 6). ConA: concanavalin A, PBMCs: peripheral blood mononuclear cells, DMSO: dimethyl sulfoxide, PSL: prednisolone, and 114: saireito.

In the PSL monotherapy group, the inhibition of PBMC proliferation was concentration-dependent compared to blank at 0.1 *μ*M and above (Figure 1, *p* < 0.05). In both PSL + 114 combination therapy groups, the inhibition of PBMC proliferation was concentration-dependent; for the PSL + TJ114 group, no significant inhibition occurred at 0.25 *μ*M or 0.5 *μ*M; however, a significant inhibition occurred from 1.0 *μ*M (Figure 1, *p*  < 0.05), while in the PSL + KR114 group, inhibition occurred at 0.1 *μ*M and higher (Figure 1,p  <  0.01). There was no significant difference between the blank and 0.4% DMSO treatments (Figure 1). There was no significant difference between the PSL monotherapy group and the PSL + 114 combination therapy group.

**Figure 2:**
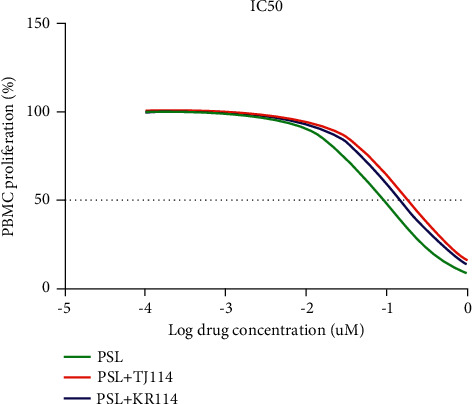
Typical dose curves of PSL, 114, and PSL + 114 for the proliferation of ConA-activated PBMCs from healthy subjects (n = 6). ConA: concanavalin A, PBMCs: peripheral blood mononuclear cells, PSL: prednisolone, and 114: saireito.

The respective IC_50_ values of the different groups were 0.0941 *μ*M for the PSL monotherapy group, 0.191 *μ*M for the PSL + TJ114 group, and 0.154 *μ*M for the PSL + KR114 group. Thus, the PSL + 114 combination therapy groups had 2.02 times (PSL + TJ114) and 1.64 times (PSL + KR114) higher IC_50_ values, respectively, than the PSL monotherapy group (Figure 2, Table 1).

### 3.2. Change in the Ratio of PBMC CD4^+^ T Cells after the Addition of PSL and 114

**Figure 3:**
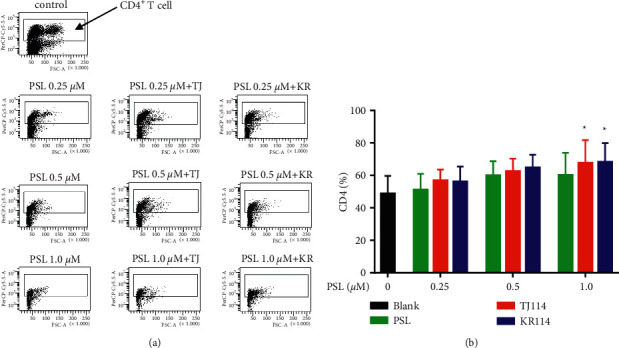
Dot plot and cell positive rate of CD4^+^ T cells in the PBMCs after PSL and 114 treatments. (a) Dot plot of CD4^+^ T cells in the PBMCs stimulated by ConA after PSL and 114 treatments (indicated in the square). Vertical axis: PerCP-Cy5-5A; horizontal axis: FSC-A. The inside of the square frame was CD4^+^ T cells, and the ratio was calculated by the number of CD4 stained cells / lymphocyte count *∗* 100. (b) Ratio of CD4^+^ T cells in PBMCs after PSL and 114 treatments. Each treatment was compared to its respective blank using Dunnett's multiple comparisons test (^*∗*^*p* < 0.05; ^*∗∗*^*p* < 0.01). The PSL monotherapy group and the PSL + 114 combination therapy groups were compared using the Tukey–Kramer multiple comparison test (mean + SD, *n* = 6). ConA: concanavalin A, PBMCs: peripheral blood mononuclear cells, PSL: prednisolone, and 114: saireito.

We compared the PSL monotherapy group to its respective blank at 1.0 *μ*M and found that the absolute number of CD4^+^ T cells in PBMCs substantially decreased, whereas the ratio of CD4^+^ T cells increased. At the same PSL concentration, the number of CD4^+^ T cells decreased in the PSL + 114 groups compared to that in the PSL monotherapy group, whereas the ratio of CD4^+^ T cells increased similarly to that in the PSL monotherapy group (Figure 3(a)). Although the results showed that the ratio of CD4^+^ T cells in the PSL monotherapy group concentration dependently increased, there was no significant increase at 1.0 *μ*M, the maximum PSL concentration used in this study.

The ratio of CD4^+^ T cells in the PSL + 114 combination therapy groups concentration dependently increased compared to the blank: 1.38 times higher in the 1.0 *μ*M PSL + TJ114 group and up to 1.40 times higher in the 1.0 *μ*M PSL + KR114 group (Figure 3(a), *p* < 0.05).

Comparing the PSL monotherapy group and the PSL + 114 combination therapy groups, the ratio of CD4^+^ T cells increased ∼1.13 times in both the 1.0 *μ*M PSL + TJ114 group and the 1.0 *μ*M PSL + KR114 group compared to that in the 1.0 *μ*M PSL group (Figure 3(b)).

Regardless of the PSL concentration used, there was no significant difference in the ratio of CD4^+^ T cells between the different saireito manufacturers of TJ114 and KR114.

### 3.3. Change in the Ratio of CD8^+^ T Cells in PBMCs after the Addition of PSL and 114

**Figure 4:**
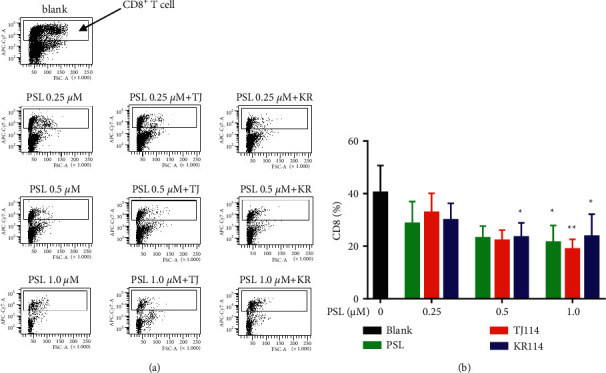
Dot plot and cell positive rate of CD8^+^ T cells in the PBMCs after PSL and 114 treatments. (a) Dot plot upon gating for CD8^+^ T cells in the PBMCs stimulated by ConA after PSL and 114 treatments (indicated in the square). Gating was conducted using APC-Cy7-A on the vertical axis and FSC-A on the horizontal. The inside of the square frame was CD8^+^ T cells, and the ratio was calculated by the number of CD8-stained cells / lymphocyte count *∗* 100. (b) Ratio of CD8^+^ T cells in PBMCs after PSL and 114 treatments. Each treatment was compared to its respective blank using Dunnett's multiple comparisons test (^*∗*^*p* < 0.05; ^*∗∗*^*p* < 0.01), and the PSL monotherapy group and the PSL + 114 combination therapy groups were compared using the Tukey–Kramer multiple comparison test (mean + SD, *n* = 6). ConA: concanavalin A, PBMCs: peripheral blood mononuclear cells, PSL: prednisolone, and 114: saireito.

The PSL monotherapy significantly decreased the absolute number of PBMCs and the proportion of CD8^+^ T cells in PBMCs at 1.0 *μ*M compared to the blanks (Figure 4(b), *p* < 0.05). In the 1.0 *μ*M PSL + 114 combination therapy groups, the number, and ratio of CD8^+^ T cells decreased similar to those in the PSL monotherapy group (Figure 4(a)). In the PSL monotherapy group, the ratio of CD8^+^ T cells concentration dependently decreased from 1.0 *μ*M PSL with ∼53% compared to the blanks (Figure 4(b),*p* < 0.05).

In the PSL + 114 combination therapy groups, the ratio of CD8^+^ T cells decreased compared to the blanks by ∼47% in the 1.0 *μ*M PSL + TJ114 group (Figure 4(b), *p* < 0.01) and up to 52% from 0.5–1.0 *μ*M in the PSL + KR combination therapy group (Figure 4(b), *p* < 0.05).

There was no significant difference in the proportion of CD8⁺ T cells between the PSL + 114 combination therapy group and the PSL monotherapy group. However, the ratio of CD8^+^ T cells tended to decrease in the PSL + TJ114 group compared to that in the PSL monotherapy group at concentrations >0.5 *μ*M (Figure 4(b)).

On the other hand, the rate of CD8^+^ T cells was similar at all concentrations in the PSL + KR combination therapy group compared to the PSL monotherapy group (Figure 4(b)).

In the comparison between the manufacturers of TJ114 and KR114, although the ratio of CD8^+^ T cells decreased more in KR114 than in TJ114 at 0.25 *μ*M PSL combinations, the ratio decreased more in TJ114 than in KR114 at the 1.0 *μ*M PSL combination (Figure 4(b)).

### 3.4. Change in the Ratio of Tfh Cells in PBMCs after the Addition of PSL and 114

**Figure 5:**
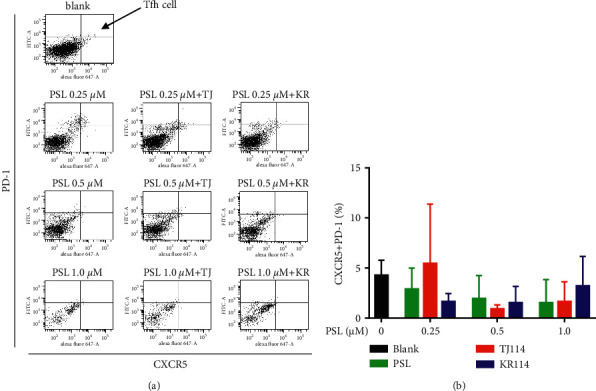
Dot plot and cell positive rate of Tfh cells in the PBMCs after PSL and 114 treatments. (a) Dot plot upon gating CXCR5^+^PD-1^+^ cells (Tfh cells) in the subcellular fractions of CD4^+^ T cells of PBMCs stimulated by ConA after PSL and 114 treatments. After gating in a PerCP-Cy5-5A-FSC-A dot plot, FITC-A in the subcellular fractions was gated on the vertical axis, and Alexa Fluor 647-A was gated on the horizontal axis. The first quadrant of the dot plot was Tfh cells, and the ratio was calculated by the number of CXCR5 and PD-1-stained cells / the number of CD4-stained cells *∗* 100. (b) Ratio of Tfh cells in PBMCs stimulated by ConA after PSL and 114 treatments. Each treatment was compared to its respective blank using Dunnett's multiple comparisons test (^*∗*^*p* < 0.05; ^*∗∗*^*p* < 0.01). The PSL monotherapy group and the PSL + 114 combination therapy groups were compared using the Tukey–Kramer multiple comparison test (mean + SD, *n* = 6). ConA: concanavalin A, PBMCs: peripheral blood mononuclear cells, PSL: prednisolone, and 114: saireito.

The absolute number of PBMCs and the ratio of Tfh cells in PBMCs were substantially decreased in the 1.0 *μ*M PSL monotherapy group compared to blanks. This was also the case in the PSL + 114 combination therapy groups (Figure 5(a)). The ratio of Tfh cells concentration dependently decreased in the PSL monotherapy group compared to that in the blank group, even though the maximum decreased ratio of ∼35% at 1.0 *μ*M was not significantly different.

The ratio of Tfh cells increased by 1.28 times in the 0.25 *μ*M PSL + TJ114 group compared to the blanks and decreased up to ∼30% at 0.5 *μ*M and above. Although the ratio of Tfh cells increased in the PSL + KR114 group, the ratio was still less than the blanks at all PSL concentrations (Figure 5(b)).

A comparison between the PSL monotherapy and the PSL + 114 combination therapy groups showed that the proportion of Tfh cells tended to decrease depending on the PSL concentration in the PSL monotherapy group; however, this decrease was not observed when combined with 114. However, there was no significant difference in the increase or decrease in the proportion of Tfh cells between the PSL monotherapy and the PSL + 114 combination therapy groups.

### 3.5. Th1, Th17, and Tfh Levels in PBMCs after the Addition of PSL and 114

**Figure 6:**
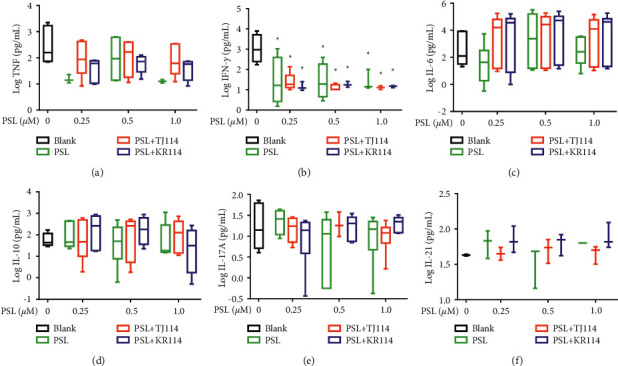
Concentration of cytokines in the PBMCs after PSL and 114 treatments. ConA, PSL, and 114 were added to PBMCs, and then the solution was incubated for 96 h. For flow cytometry, beads were used to analyze the concentration of TNF, IFN-*γ*, IL-6, IL-10, IL-17A, and IL-21 in the culture supernatant. Each treatment was compared to its respective blank using Dunnett's multiple comparisons test (^*∗*^*p* < 0.05; ^*∗∗*^*p* < 0.01). The PSL monotherapy group and the PSL + 114 combination therapy groups were compared using the Tukey–Kramer multiple comparison test (median + SD, *n* = 6). ConA: concanavalin A, PBMCs: peripheral blood mononuclear cells, PSL: prednisolone, and 114: saireito.

After treating PBMCs with PSL and 114, we measured the concentrations of TNF (A), IFN-*γ* (B), IL-6 (C), IL-10 (D), IL-17A (E), and IL-21 (F) (Figure 6).

PSL monotherapy reduced the TNF concentration to 5.43 pg/mL at the maximum PSL concentration of 1.0 *μ*M compared to blank (131 pg/mL), albeit with no significant difference. Although the TNF concentration decreased in the PSL + TJ114 group by 70.1% at the maximum PSL concentration of 1.0 *μ*M compared to the blank, the difference was not significant. In comparison to the blank, the PSL + KR114 group showed a 78.3% reduction at the maximum PSL concentration of 1.0 *μ*M; however, no significant difference was found. There was no significant difference in the TNF concentration between the PSL monotherapy and PSL + 114 combination therapy groups at similar PSL concentrations (Figure 6(a)).

Compared to the median IFN-*γ* concentration in the blanks (1049 pg/mL), both PSL monotherapy and PSL + 114 combination therapy significantly decreased the IFN-*γ* concentration at 0.25 *μ*M PSL and above (*p* < 0.05). At the maximum PSL concentration of 1.0 *μ*M, the IFN-*γ* concentration was lowered by 99.42% in the PSL monotherapy group, by 99.51% in the PSL + TJ114 group, and by 99.40% in the PSL + KR114 group compared to the blanks. There was no significant difference in IFN-*γ* concentrations at similar PSL concentrations between the PSL monotherapy and PSL + 114 combination therapy groups (Figure 6(b)).

Furthermore, PSL monotherapy increased IL-6 concentration by 1.83-fold at 1.0 *μ*M compared to the median blank (188 pg/mL), although the change was not concentration-dependent. The PSL + 114 combination therapy increased IL-6 concentration at 1.0 *μ*M by 66.2-fold in the PSL + TJ114 group and up to 219-fold in the PSL + KR114 group compared to the blanks (Figure 6(c)).

Moreover, the PSL monotherapy decreased IL-10 concentration compared to the blanks, though it was not concentration-dependent. At 1.0 *μ*M PSL, the median IL-10 concentration for blanks (40.2 pg/mL) decreased by 57.1%. The PSL + TJ114 combination therapy increased the IL-10 concentration compared to the blanks, though it was also not concentration-dependent. At 0.5 *μ*M PSL, the IL-10 concentration increased by 3.42-fold compared with blanks. The PSL + KR114 combination therapy increased IL-10 concentration compared with blanks, although the change was not concentration-dependent. At 1.0 *μ*M PSL, the IL-10 concentration increased by 1.10-fold compared with the blanks. The PSL + 114 combination therapy group tended to induce a higher increase in IL-10 concentration than the PSL monotherapy group (Figure 6(d)).

The 1.0 *μ*M PSL monotherapy decreased the median value of IL-17A by 0.31% compared to that of the blank (14.8 pg/mL). On the contrary, the median value of IL-17A decreased by 18.9% in the PSL + TJ114 combination therapy group and increased by 1.18-fold in the PSL + KR114 combination therapy group (Figure 6(e)).

The PSL monotherapy concentration dependently decreased the IL-21 concentration, which was not observed with PSL + 114 combination therapy (Figure 6(f)).

## 4. Discussion

This study examined the immunoregulatory action of PSL, 114, and PSL + 114 using PBMCs from healthy adults. Altogether, we examined their effects on PBMC proliferation, the ratio of CD4^+^, CD8^+^ T cells, and Tfh cells, respectively, and the production of TNF, IFN-*γ*, IL-6, IL-10, IL-17A, and IL-21. In summary, the proliferation of PBMCs was dose dependently suppressed in both the PSL and PSL + 114 groups. Combination therapy increased the IC_50_ in the PSL group by 1.5∼2-fold in the PSL + 114 groups. Lastly, the ratio of CD4^+^ T cells increased in both the PSL + 114 groups compared to that in the PSL group without affecting the ratio of CD8^+^ T cells and Tfh cells.

Studies have reported that PSL inhibits the proliferation of human PBMCs [19]. In line with this, we also showed a concentration-dependent inhibition of PBMC proliferation by 0.1 *μ*M and above PSL monotherapy. However, when high doses of PSL were used in combination with 114, the results did not differ significantly from those of PSL alone at an equal concentration; that is, no additional inhibition of PBMC proliferation was observed. Since this study used 114 at a concentration of 300 *μ*g/mL, the clinically relevant dose found in blood [18], it has been suggested that, at high concentrations of PSL, the action of PSL is strongly expressed and does not cause a synergistic effect on the inhibition of PBMC proliferation. In contrast, it was suggested that a remarkable synergistic effect was exhibited on the suppression of PBMC proliferation, although there was no significant difference in low-concentration PSL. In this study, the PSL + 114 combination therapy showed greater inhibition of PBMC proliferation than PSL monotherapy at 0.0001 *μ*g/mL, which implied that extremely low concentrations of PSL could possibly demonstrate synergistic immunosuppressive action in combination with 114. Depending on these findings, it is expected that if the dose of PSL is reduced due to side effects, it can be administered in combination with 114 to avoid impairing the therapeutic effect of PSL. On the other hand, the action of PSL at higher concentrations might dwarf the effects of 114. Studies have reported that combining 114 and fluocinolone acetonide (FA) impairs the leukocyte proliferation suppressive action of FA more than 114 by itself [20]; while this is a different type of steroid, it still suggests that 114 can diminish the effect of corticosteroids on the immune system. In this regard, 114 can potentially diminish the inhibitory action of PSL on PBMCs, as we found that the PSL + 114 combination therapy increased the IC_50_ to 1.6–2.0 times that of PSL monotherapy.

We also examined the fluctuation in T-cell subsets and the change in the cytokines they produce because cytokine production can result in the activation of T cells, a possible reason for the increase in PBMCs based on the type of 114 combinations used. Toward this goal, we examined the fluctuation of CD4^+^ and CD8^+^ T cells, as well as Tfh cells in PBMCs when PSL was used alone and in combination with 114. The CD4^+^ T-cell ratio increased to 60.1% at 1.0 *μ*M in the PSL monotherapy group compared to the blank (CD4^+^ T-cell ratio: 48.7%). At the same concentration, the ratio of CD4^+^ T cells significantly increased to 67.6% and 68.1% in the PSL + TJ114 and PSL + KR114 combination therapy groups, respectively. Previously, 114 has been reported to increase the ratio of CD4^+^ T cells in PBMCs [21]. In line with this study, we also attribute the increase in the ratio of CD4^+^ T cells observed in the PSL+114 combination therapy groups to the action of 114.

In autoimmune diseases, CD8^+^ T cells are related to disease activity through insufficient regulation of the secretion of inflammatory cytokines, differentiation bias, and the inappropriate induction of apoptosis of target cells [22]. Therefore, decreasing CD8^+^ T cells is important for improving disease activity. The ratio of CD8^+^ T cells decreased in a concentration-dependent manner in the PSL monotherapy group with a significant decrease at 1.0 *μ*M. However, combining 114 with PSL did not affect the decrease in the ratio of CD8^+^ T cells observed when using PSL alone. There is a concern that macromolecules that include 114 might stimulate the proliferation of CD8^+^ T cells in autoimmune diseases, similar to how ginsenoside Rg1 included in ginseng (a component of 114) has been reported to increase the ratio of CD8^+^ T cells *in vivo* [23]. In contrast, the results of this study showed that 114 did not affect the increase in the ratio of CD8^+^ T cells when used in combination with PSL, suggesting that 300 *μ*g/mL 114 is a safe dosage that does not elevate the ratio of CD8^+^ T cells when used in combination with PSL *in vitro*.

An increase in Tfh cells is reported to be related to a worsening of the clinical state of autoimmune diseases [17], though the combined effect of PSL and 114 on Tfh cells is unclear. The results of this study showed a slight decrease in the ratio of Tfh cells in the PSL monotherapy group, though not significantly different, and it remains unclear whether the combined use with 114 can possibly increase Tfh cells. To date, there are no reports of the effect of PSL alone on Tfh cells *in vitro*; this study showed that PSL can suppress the expression of PD-1 and CXCR5, suggesting that PSL can decrease the ratio of Tfh cells. The above results clarified that the combination of 114 with PSL can increase the ratio of CD4^+^ T cells without affecting CD8^+^ T cells. Therefore, the increase in IC_50_ in PSL when combined with 114 may potentially increase CD4^+^ T cells. On the other hand, this study also suggests that Tfh cells can be increased when PSL is combined with 114.

Next, we examined the variation in the cytokines produced by each T-cell subset. As there was no significant difference in TNF and IFN-*γ* levels at any PSL concentration in the PSL monotherapy or PSL + 114 combination therapy groups, this study was unable to show whether 114 has a definitive effect on Th1-produced cytokine inhibition. Although it has been reported that 114 does not affect TNF or IFN-*γ* production in human PBMCs at 100 *μ*g/mL [24], this study further suggests that even concentrations of 300 *μ*g/mL do not affect these parameters. In addition, although the production of Th2-produced cytokines was inhibited in the PSL monotherapy group, it increased in the PSL + 114 combination therapy groups. IL-6 plays an important role in the differentiation of Th1 and Th2 cells: it reportedly inhibits the differentiation of Th1 and facilitates the differentiation of Th2 [25]. Therefore, the CD4^+^ T cells that were increased by the combined use of 114 could possibly be Th2. As a result, using PSL in combination with 114 increased the concentration of Th2 cytokines, such as IL-6, suggesting that this combination could potentially restructure Th1 and Th2 composition (toward Th2 dominant) and improve the clinical state of Th1-mediated autoimmune diseases. In contrast, this also suggests that IL-6 could potentially worsen the clinical state of Th2-autoimmune diseases, such as RA. Neither PSL monotherapy nor PSL + 114 combination therapy altered IL-17 production compared to the blanks. In addition, there was no significant difference in IL-17 levels between the treatment groups, suggesting that PSL and 114 did not affect IL-17 production from Th17 cells. Interestingly, the median IL-21 level was 0 pg/mL, which could mean IL-21 is only produced in response to autoantibodies in autoimmune diseases, while the level of IL-21 produced by healthy adults is below the detection limit.

The major difference between TJ114 and KR114 is that the latter is composed of large amounts of Alismataceae, *Polyporus*, *Poria cocos*, and cassia bark, and the former includes *Atractylodes Rhizome*, whereas the latter includes *Rhizoma Atractylodis Macrocephalae*. Although Kampo medicines with similar names have different natural medicines and contents, such as the two mentioned above, there are few studies that explicitly compare them. In this study, there was no significant difference between TJ114 and KR114 in terms of their inhibition of PBMC proliferation, change in the ratio of CD4^+^ T cells, CD8^+^ T cells, and Tfh cells, or the production of TNF, IFN-*γ*, IL-6, IL-10, IL-17A, or IL-21. In addition, at the concentration of 300 *μ*g/mL used in this study, there was no difference in action of the products between manufacturers when used in combination with PSL, and there was no evidence sufficient for different manufacturers to use according to the disease.

This study is not without limitations. One limitation is the use of PBMCs from healthy adults, which have a lower absolute quantity of autoimmune disease-specific T cells and cytokines, such as Tfh cells, IL-17, and IL-21, compared to PBMCs from adults with an autoimmune disease. In addition, in the human body, drugs undergo pharmacokinetic changes, such as absorption, distribution, metabolism, and excretion. However, in the present study, only *in vitro* analyses were performed, and thus, further *in vivo* analyses are required to better understand and assess the mechanisms of 114. In addition, regarding the difference in the action of TJ and KR on PBMC, it is considered that it is due to the difference in the constituent crude drugs, but sufficient information on the effect of each crude drug on PBMC has not been obtained.

## 5. Conclusions

The results of this study suggest that 114 has the potential to diminish the immunosuppressive action of PSL as it increased the IC_50_ of PSL without strengthening the immunosuppressive action. Furthermore, although 114 was previously reported to have a steroid dose-reducing action, this study revealed that it was not due to an enhanced immunosuppressive effect on T cells and cytokines when concomitantly used at clinical doses. We hypothesize that the anti-inflammatory action of 114 reduces the dosage of PSL when used in combination. In addition, PSL + 114 combination therapy increased the ratio of CD4^+^ T cells and increased Th2-produced cytokines compared to PSL monotherapy. Therefore, 114 combination therapy could potentially reduce the required dose of steroids used in Th1-mediated autoimmune diseases by restoring the T-cell balance to Th2. Therefore, it is necessary to monitor these clinical parameters when using 114 in combination with PSL in treating autoimmune diseases.

## Figures and Tables

**Table 1:** IC_50_ for the PSL monotherapy and PSL + 114 combination therapy groups.

IC_50_ (*μ*M)		
PSL	PSL + TJ114	PSL + KR114
0.0947	0.191	0.154

IC_50_ was calculated using GraphPad Prism v8.0.

## Data Availability

The data used to support the findings of this study are available from the corresponding author upon request.
